# Fine-Grained Radio Frequency Fingerprint Recognition Network Based on Attention Mechanism

**DOI:** 10.3390/e26010029

**Published:** 2023-12-27

**Authors:** Yulan Zhang, Jun Hu, Rundong Jiang, Zengrong Lin, Zengping Chen

**Affiliations:** School of Electronics and Communication Engineering, Sun Yat-sen University, Shenzhen 518107, China; zhangylan23@mail2.sysu.edu.cn (Y.Z.); jiangrd3@mail2.sysu.edu.cn (R.J.); linzr9@mail2.sysu.edu.cn (Z.L.); chenzengp@mail.sysu.edu.cn (Z.C.)

**Keywords:** RF fingerprint, fine-grained classification, internet of things, attention mechanism

## Abstract

With the rapid development of the internet of things (IoT), hundreds of millions of IoT devices, such as smart home appliances, intelligent-connected vehicles, and wearable devices, have been connected to the network. The open nature of IoT makes it vulnerable to cybersecurity threats. Traditional cryptography-based encryption methods are not suitable for IoT due to their complexity and high communication overhead requirements. By contrast, RF-fingerprint-based recognition is promising because it is rooted in the inherent non-reproducible hardware defects of the transmitter. However, it still faces the challenges of low inter-class variation and large intra-class variation among RF fingerprints. Inspired by fine-grained recognition in computer vision, we propose a fine-grained RF fingerprint recognition network (FGRFNet) in this article. The network consists of a top-down feature pathway hierarchy to generate pyramidal features, attention modules to locate discriminative regions, and a fusion module to adaptively integrate features from different scales. Experiments demonstrate that the proposed FGRFNet achieves recognition accuracies of 89.8% on 100 ADS-B devices, 99.5% on 54 Zigbee devices, and 83.0% on 25 LoRa devices.

## 1. Introduction

The Internet of things (IoT) [1] consists of many context-aware products and technologies, ranging from analog and digital sensors to global positioning systems (GPSs), radio frequency identification devices (RFIDs), near field communication (NFC) sensors, weather detectors and emergency alarms, etc. The sector has been growing rapidly and is greatly changing our daily lives and industries.

Compared with a traditional network, decentralization and heterogeneity are the biggest features of IoT. These also introduce security issues, such as passive eavesdropping, identity spoofing attacks, replay attacks, and so on. Authentication is gaining increased attention, especially in the context of wireless communication within the IoT, as the open nature of the wireless medium exposes additional security vulnerabilities [2].

Traditional authentication employs cryptography-based methods to verify whether a message is generated by a trusted source or not [3]. Generally, these methods require high communication overhead and complexity at the application layer due to complicated operations such as encryption and decryption, key sharing, and management [4]. Thus, they are not affordable for common IoT devices with limited computation and power resources. By contrast, physical-layer authentication is believed to be a kind of secure and terminal cost-free method for IoT systems.

Physical-layer identification is the process of fingerprinting a wireless device by extracting features of hardware imperfections [5,6]. These hardware imperfections are referred to as RF fingerprints, which are, similar to human fingerprints, intrinsic and unique. Moreover, they are independent of the modulation mode, remain unaffected by the environment, and introduce subtle differences in signals. Even for wireless devices produced by the same manufacturer with the same components in the same batch, differences in RF fingerprints still exist.

In the past few decades, many efforts have been made for RF-fingerprint-based recognition, and they can be classified into two categories: machine-learning-based [7,8,9,10,11,12,13] and deep-learning-based methods [14,15,16,17,18,19,20,21,22]. The former requires manually extracting the designed and specific RF fingerprint features from the RF signal and then using a classifier to identify the device. The latter employs deep neural networks to automatically extract features and identify different devices and achieves higher accuracy [23]. However, deep-learning-based methods are faced with two main challenges:

- Because the differences between emitters are fairly subtle, especially for transmitters of the same model produced in the same batch by the same manufacturer, the inter-class variation in RF fingerprints is low.
- RF fingerprints are unintentional and weak modulations that are incidental to the transmitted signal. They are susceptible to complex channel conditions, multi-path effects, and environmental noise. As a result, large intra-class variation exists in the RF fingerprints of the same device.

In this work, we attempt to deal with the above issues by combining the fine-grained recognition used in computer vision with RF-fingerprint-based recognition. Fine-grained recognition can divide a coarse-grained meta-category into more-detailed subcategories: distinguishing subcategories with low inter-class variation and high intra-class variation. Compared with general image classification, fine-grained image recognition can complete more difficult classification tasks.

Inspired by this, we propose a fine-grained RF fingerprint recognition network, which includes an adaptive spatial feature fusion module integrating both the high-level semantic and the low-level detailed information as well as an attention module to refine feature maps. As the structure of a CNN goes deeper, the neurons in high layers respond strongly to entire images and are rich in semantics, but they inevitably lose detailed information (e.g., color, edge junctions, texture patterns) from small discriminative regions [24]. To extract finer-grained features, we use ResNet [25] as the backbone and generate the pyramidal features by establishing a top-down feature pathway hierarchy on a basic CNN. In addition, we also utilize an attention mechanism to enhance the feature representation and to accurately locate discriminative regions. The contributions of this paper are as follows:

- We propose a fine-grained RF fingerprint recognition network that generates pyramidal features by establishing a top-down feature pathway hierarchy on the basic network. Those feature maps are further refined by the attention module and are integrated to learn fine-grained features. To the best of our knowledge, we are the first to combine fine-grained recognition with RF fingerprint identification tasks.
- We combine spatial attention and channel attention and propose a novel second-order attention module. It sequentially infers the attention map along two independent dimensions: channel and space. Subsequently, the attention map is multiplied by the input feature map to localize significant regions, enabling the learning of fine-grained and discriminative features.
- In contrast to the conventional approach of element-wise summation or concatenation used in previous studies to fuse multi-scale features, we propose the adaptive spatial feature fusion module (ASFFM) to adaptively integrate features from different scales. This allows for the fusion of high-level semantic features and low-level detailed features: ensuring that the fine-grained details are not lost and thus facilitating more accurate fine-grained recognition.
- We conduct extensive experiments on three challenging datasets including 100 ADS-B devices, 54 Zigbee devices, and 25 LoRa devices and achieve superior performance over the state-of-the-art approaches. A visualization and comprehensive experiments are further conducted to draw insights about our method.

The remainder of this paper is organized as follows. A literature review of RF-fingerprint-based identification and fine-grained recognition is presented in Section 2. A signal model is described in Section 3, and the proposed method is elaborated upon in Section 4. Experiments and the relevant discussion are presented in Section 5, while conclusions are drawn in Section 6.

## 2. Related Works

In this section, we provide a brief review of RF-fingerprint-based recognition in IoT and fine-grained recognition in deep learning.

### 2.1. RF Fingerprint Recognition

Prior to 2018, research on RF fingerprints focused on machine-learning-based algorithms, such as support vector machine (SVM), to identify the identity of each mobile device. After 2018, research using deep learning gradually emerged.

#### 2.1.1. Machine Learning

The machine-learning-based approach mainly consists of two stages: extraction of manually designed RF fingerprint features and classification. Manually designed RF fingerprints can be divided into transient-state and steady-state features according to the signal state. Transient-based RF fingerprinting techniques use the transition from the turn-off to the turn-on of a transmitter before the transmission of the actual signal [5]. Hall et al. [7] used signals captured from Bluetooth transceivers and extracted instantaneous amplitude, phase, in-phase, quadrature, instantaneous power, and discrete wavelet transform (DWT) coefficients as RF fingerprints to classify 10 different Bluetooth devices. Yuan et al. [8] employed the transient signal’s time–frequency–energy distribution obtained by the Hilbert–Huang transform (HHT) to construct feature vectors and used SVM to classify the devices. These approaches require accurate transient extraction (start point and duration) before feature extraction and identification [9], which limits their real application.

Steady-state features are extracted from a signal when the transmitter is in a stable working state, making these signals more enduring and easier to obtain. The authors of [10] proposed a radiometric-signature-based device identifier named the passive radiometric device identification system (PARADIS). It takes into account the influence of various non-ideal characteristics of the device on the modulated signal, including I/Q channel offset, frequency offset, amplitude limit, etc., and successfully realized the identification of 138 wireless network cards with QPSK modulation by SVM. Although the overall recognition accuracy of PARADIS is high, it is easily affected by ambient noise. The work in [11] proposed a method for identifying different devices by extracting the RF fingerprint from the modulation shape and spectral features acquired from RFID transponders. This method is computationally efficient and can form compact fingerprints. But the fingerprints extracted from the same device vary when acquired from a long distance (e.g., more than 1 m). Peng et al. [12] employed the DCTF to capture the time-varying modulation error of Zigbee devices and developed a low-overhead classifier to identify 54 Zigbee devices. DCTF can extract features (e.g., I/Q channel deviations and non-linear variations) caused by hardware defects and eliminate the influence of the carrier frequency offset. Williams et al. [13] proposed a physical-layer authentication (PLA) scheme based on the features of the amplitude, phase, and frequency, and used a Fisher-based classifier to perform an authentication decision. The lowest recognition accuracy for three devices of the same type was only 83.25%, which demonstrates that the differences between the extracted RF fingerprints are a little subtle.

#### 2.1.2. Deep Learning

Sankhe et al. in [14], designed a convolutional neural network (CNN) with two convolutional layers and two fully connected layers to train on RF fingerprint data from 16 bit-similar USRP X310 SDRs. The network structure is relatively simple, which limits further improvement to its accuracy. Some works used general architectures in computer vision classification to classify RF fingerprints, such as 1D modified versions of AlexNet (AlexNet1D) and ResNet-50 (ResNet1D) [15,16], VGG-16 [17], attention mechanism [18], and so on. The researchers modified the convolution kernel to be one-dimensional to make it suitable for the 1D RF signals without considering a network architecture specifically applicable to the existing challenges of RF fingerprint recognition. Zhang et al. [19] proposed an adaptive RF fingerprint fusion network to extract and adaptively fuse multiple RF fingerprints in a data-driven manner that is robust to channel and SNR variations. Generative adversarial nets (GANs) [20] have also been applied to implement adversarial learning for identifying RF transmitters. Most of the neural-network-based approaches mentioned above primarily utilize monomodal information from the time or frequency domain. However, they overlook the potential benefits of integrating multimodal information from multiple transformation domains, which can provide complementary processing gains. Consequently, some works have adopted multimodal methods that leverage neural networks to learn features from different modalities with the aim of enhancing recognition accuracy. An et al. [21] proposed a novel approach called the series constellation multimodal feature network (SC-MFNet) for identifying the modulation types of MIMO-OFDM subcarriers. Qi et al. [22] employed the waveform–spectrum multimodal fusion (WSMF) method combined with a deep residual network (ResNet) to implement an automatic modulation classification (AMC) algorithm. By extracting features from multimodal information using ResNet and employing a feature fusion strategy, the multimodal features of the signals are merged to obtain more discriminative characteristics.

### 2.2. Fine-Grained Recognition

Fine-grained recognition is designed to deal with objects that belong to multiple subordinate categories of the same meta-category (such as the Alaskan and Husky subcategories in the dog category) and to discriminate objects that are highly similar in overall appearance but differ in fine-grained features. The majority of the fine-grained community focuses on two streams: (1) part localization methods and (2) feature encoding methods. Most part-based methods focus on learning explicit part localizations by detection/segmentation [24,26,27] or attention learning. The methods for detection or segmentation are typically two-stage: first, region proposals are generated, and then, the proposed regions are classified. Two-stage networks are usually time-consuming and slow.

Attention-based methods are normally one-stage and end-to-end and are widely applied in fine-grained recognition. TASN [28] is a trilinear attention sampling network that learns fine-grained details from hundreds of part proposals and efficiently distills the learned features into a single CNN. API-Net [29] learns unique features by simultaneously training the differences between a pair of similar images through the attention mechanism. ACNet [30] presents an attention-based convolutional binary neural tree to facilitate coarse-to-fine hierarchical fine-grained feature learning. The main innovation of this method is the combination of a binary tree and an attention mechanism as well as the application of an attention transformer. In this way, the network can learn the coarse-to-fine hierarchical discriminative features.

For feature encoding methods, MC-Loss [31] introduced a single loss function to learn subtle details without the requirement of an overly complicated network design. MC-Loss can easily be integrated into any backbone network with low complexity and stable performance. However, when tackling complex datasets, the improved accuracy is limited. Bilinear-CNN [32] encodes the second-order statistical information of convolutional activations as image features by multiplying the feature maps extracted by two CNNs and then performing pooling. It significantly improved fine-grained recognition, but the relevant covariance leads to a large increase in the number of associated parameters.

## 3. Signal Model

We take the DCTF representation in [33] as the input for FGRFNet. This first requires obtaining a differential form of the transmitted RF signals and then converting the time-domain complex signals into a 2D image.

### 3.1. RF Signal

For simplicity, we consider the ideal RF signal as follows:

(1) $$x\left(t\right)=x_{I}\left(t\right)+jx_{Q}\left(t\right)$$

and the transmitted RF signal can be written as

(2) $$x\left(t\right)=\left(\left(\beta_{I}x_{I}\left(t\right)+\alpha_{I}\right)+j\left(\beta_{Q}x_{Q}\left(t\right)+\alpha_{Q}\right)\right)\cdot e^{-j2\pi f_{c}^{t}t}$$

where $x_{I}\left(t\right)$ and $x_{Q}\left(t\right)$ represent the real and imaginary parts of the RF signal $x\left(t\right)$: namely, the I-channel part and the Q-channel part, respectively; $\beta_{I}$ and $\beta_{Q}$ are the respective I/Q gain imbalances; $\alpha_{I}$ and $\alpha_{Q}$ are the respective DC offsets on the I/Q channel; and $f_{c}^{t}$ is the carrier frequency of the transmitter.

The I/Q imbalance and DC offset are typical RF fingerprints and are difficult to extract directly in the time domain because they are mixed with modulation and encoding. Assuming that the channel and receiver are ideal, the received signal can be demodulated as:

(3) $$y\left(t\right)=x\left(t\right)\cdot e^{j2\pi f_{c}^{r}t}+n\left(t\right)=y_{I}\left(t\right)+jy_{Q}\left(t\right)$$

where $y_{I}\left(t\right)$ and $y_{Q}\left(t\right)$ are, respectively, the real and imaginary parts of the I/Q channel of the received signal $y\left(t\right)$, $f_{c}^{r}$ is the carrier frequency of the receiver, and $n\left(t\right)$ is the noise.

Generally, there exists a carrier frequency offset (CFO) $\theta =f_{c}^{r}-f_{c}^{t}$, which is mainly caused by the crystal oscillator mismatch between the receiver and the transmitter or by the Doppler frequency shift.

### 3.2. DCTF

As pointed out in [33], DCTF is generated by converting the complex time-domain differential RF signal into a 2D constellation figure.

#### 3.2.1. Differential Signal

The complex time-domain differential RF signal is obtained by:

(4) $$\begin{array}{cc} d\left(t\right) & =\left(y_{I}\left(t\right)+jy_{Q}\left(t+\varepsilon \right)\right)\cdot {\left(y_{I}\left(t+\lambda \right)+jy_{Q}\left(t+\lambda +\varepsilon \right)\right)}^{*}\\ \; & =\left(\left(\beta_{I}x_{I}\left(t\right)+\alpha_{I}\right)+j\left(\beta_{Q}x_{Q}\left(t+\varepsilon \right)+\alpha_{Q}\right)\right)\cdot e^{j2\pi \theta t}\\ \; & \cdot \left(\left(\beta_{I}x_{I}\left(t+\lambda \right)+\alpha_{I}\right)-j\left(\beta_{Q}x_{Q}\left(t+\lambda +\varepsilon \right)+\alpha_{Q}\right)\right)\\ \; & \cdot e^{-j2\pi \theta \left(t+\lambda \right)}\\ \; & =\left(\left(\beta_{I}x_{I}\left(t\right)+\alpha_{I}\right)\cdot \left(\beta_{I}x_{I}\left(t+\lambda \right)+\alpha_{I}\right)\right.\\ \; & \left.-j\left(\beta_{I}x_{I}\left(t\right)+\alpha_{I}\right)\cdot \left(\beta_{Q}x_{Q}\left(t+\lambda +\varepsilon \right)+\alpha_{Q}\right)\right.\\ \; & \left.+j\left(\beta_{Q}x_{Q}\left(t+\varepsilon \right)+\alpha_{Q}\right)\cdot \left(\beta_{I}x_{I}\left(t+\lambda \right)+\alpha_{I}\right)\right.\\ \; & \left.+\left(\beta_{Q}x_{Q}\left(t+\varepsilon \right)+\alpha_{Q}\right)\cdot \left(\beta_{Q}x_{Q}\left(t+\lambda +\varepsilon \right)+\alpha_{Q}\right)\right)\\ \; & \cdot e^{-j2\pi \theta \lambda }\\ \; & =d_{I}\left(t\right)+jd_{Q}\left(t\right) \end{array}$$

where $d_{I}\left(t\right)$ and $d_{Q}\left(t\right)$ are the real and imaginary parts of the differential signal, respectively; λ is the differential time interval; ε is the introduced I/Q phase mismatch, which is used to amplify the fingerprint features; and ${\left(\cdot \right)}^{*}$ represents the conjugation operation.

Note that the CFO introduces a constant phase rotation factor $e^{-j2\pi \theta \lambda }$ in $d\left(t\right)$ and does not change with the position of the sample point.

#### 3.2.2. Constellation Figure

In digital communication, digital signals are often represented by points on the complex plane to visually represent the relationship between different signals, which is precisely the constellation figure. In order to display the characteristics of RF fingerprints more intuitively, DCTF counts the distribution density of $d\left(t\right)$ over the I/Q channels on the constellation figure.

We divide the entire constellation figure into N×N sub-regions. A density matrix Φ is built to count the number of distribution points in each sub-region. $\Phi_{i,j}$ represents the number of points in the sub-region $\left(i,j\right)$ on the constellation figure. Finally, each element of Φ is normalized in the range from 0 to 255, and the normalized Φ is just the DCTF.

#### 3.2.3. Zigbee Examples

**Figure 1:**
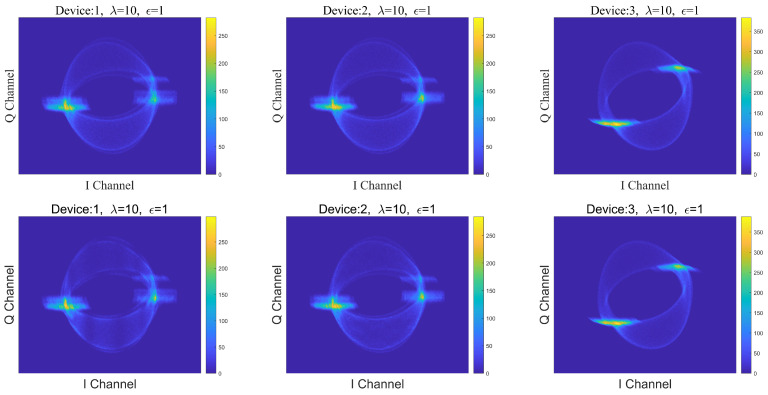
Examples of the DCTFs generated by three real Zigbee devices.

Some DCTF examples of Zigbee devices are shown in Figure 1, where each row represents the DCTFs from different devices, and each column represents the DCTFs of different signals emitted by the same device.

It can be seen that the differences in DCTFs between Device 1 and 2 are subtle, and this indicates the low inter-class variation. But the two DCTFs from Device 1 in the first column can be distinguished by the naked eye, and this indicates the large intra-class variation of the same emitter.

Since the Zigbee signal is modulated by offset quadrature phase-shift keying (OQPSK), $x_{I}\left(n\right)=\left\{\pm 1\right\}$ and $x_{Q}\left(n\right)=\left\{\pm 1\right\}$. By substituting $x_{I}$ and $x_{Q}$ into (4), it can be concluded that $d\left(t\right)$ has the following possibilities:

(5) $$\begin{array}{cc} d\left(t\right) & =\left(\beta_{I}^{2}m_{1}+\beta_{Q}^{2}m_{2}+\alpha_{I}\beta_{I}n_{1}+\alpha_{Q}\beta_{Q}n_{2}+\alpha_{I}^{2}+\alpha_{Q}^{2}\right)\\ \; & +j\left(\beta_{I}\beta_{Q}n_{3}+\alpha_{I}\beta_{Q}n_{4}+\alpha_{Q}\beta_{I}n_{5}\right) \end{array}$$

where $m_{j}=\left\{\pm 1\right\},j=1,2$, $n_{i}=\left\{0,\pm 2\right\},i=1,2,\cdots ,5$. As can be deduced from Equation (5), the locations of the DCTF gathering center can effectively reflect the inherent defects of the transmitter brought by the I/Q modulator: namely, the RF fingerprint. In addition, DCTF can also suggest the inherent non-linear characteristics of the power amplifier. The input signal may be distorted after the power amplifier; this is mainly manifested as constellation point deviation and dispersion.

In general, DCTF can extract I/Q channel deviations, non-linear variations, etc., and does not require any synchronization information. Its aggregation points reflect the statistical mean of $d\left(t\right)$, and the distribution reflects the variance caused by noise and random wireless channels [33].

## 4. The Proposed Method

Most of the previous works simply migrated networks commonly used in deep learning to the task of RF fingerprint recognition, and their performance may not be that outstanding. In our experiments, we observed that devices exhibit small inter-class differences and large intra-class differences in RF fingerprints. This is a challenge that was not pointed out and addressed by previous methods. Therefore, targeting this challenge in RF fingerprint recognition, we specifically propose FGRFNet. Unlike previous research, we propose a second-order attention module. In contrast to earlier attention-based approaches, our method captures long-range dependencies and finely models complex relationships between elements, enhancing the model’s expressive power. Furthermore, we propose an adaptive spatial feature fusion module. Previous works typically used element-wise summation or concatenation to fuse multi-scale features, implying equal weighting for different features, which is evidently unreasonable. The adaptive fusion module we propose autonomously learns fusion weights for each pixel in feature maps of different scales, fusing high-level semantic features with low-level details. This enables the network to extract discriminative fine-grained features for distinguishing subtly different devices.

### 4.1. Data Sample

We found that if the original complex I/Q signal is directly fed into the neural network, the neural network cannot learn the true RF fingerprint. For example, in ADS-B data, it will learn the international civil aviation organization (ICAO) address code that represents the aircraft’s identity.

Therefore, we directly generate the DCTF representations of the signals and feed the DCTFs into the fine-grained network. Notably, each transmitter has a unique label. Different devices of the same batch and type from the same manufacturer have different labels, and this conforms to the scope of the fine-grained classification. In the subsequent experiments, we visually present the learned RF fingerprints in a heat map.

### 4.2. FGRFNet

**Figure 2:**
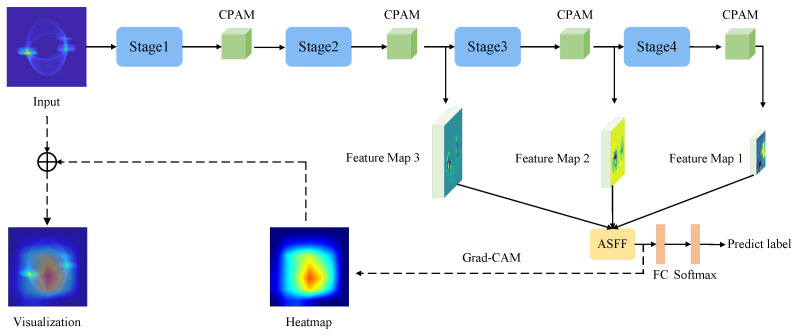
The structure of FGRFNet.

An overview of the proposed method for fine-grained RF fingerprint recognition is shown in Figure 2. It consists of four modules: namely, the backbone network, the channel and positional attention module (CPAM), the adaptive spatial feature fusion module (ASFFM), and the classifier.

Firstly, the network takes as input a DCTF, which is fed into convolutional layers to extract pyramidal feature maps. Then, these feature maps are further transformed into pyramidal enhanced discriminative feature representations by the CPAM, following a bottom-up pathway. Once the attention feature pyramid has been obtained from the raw input, an ASFFM integrates the low-level detailed information with high-level semantic feature representations. Finally, a group of probability scores over the fused features to fine-grained categories are predicted by fully connected and softmax layers.

The proposed FGRFNet is optimized for convergence by learning a cross-entropy loss. Note that it can be trained end-to-end, and the framework is flexible on CNN backbone structures.

### 4.3. CPAM

**Figure 3:**
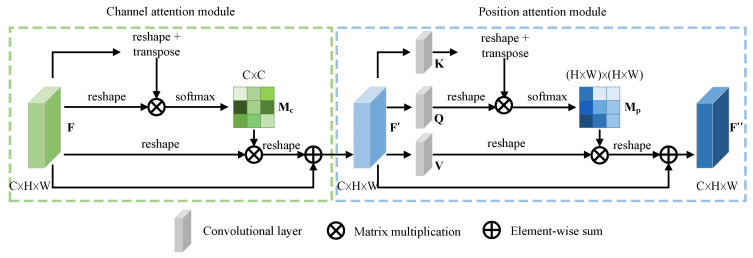
The structure of CPAM.

Inspired by [34], we combine spatial attention and channel attention and propose a novel second-order attention module. As illustrated in Figure 3, given a feature map F as input, CPAM sequentially infers a channel attention matrix $M_{c}\in R^{C\times C}$ and a spatial attention matrix $M_{p}\in R^{\left(H\times W\right)\times \left(H\times W\right)}$, where $M_{c}$ and $M_{p}$ respectively model the channel and spatial relationships between any two pixels of the features.

**Channel Attention Module:** As pointed out in [28,35], a convolutional feature channel often corresponds to a certain type of visual pattern. By clustering spatially correlated channels for which the peak responses appear in neighboring locations, the fine-grained part-feature representation can be improved. Therefore, we build a channel attention module (CAM) to explicitly model the channel dependency.

Considering an input image, we first extract the feature maps by a base network that includes a series of convolution and pooling operations. Assume that the dimension of the feature map F is C×H×W, where *C*, *H*, and *W* denote the channel number, height, and width, respectively. We first reshape F into a matrix with a shape of C×N, N=H×W, and denote it as $F_{r}={\left[f_{1},\ldots ,f_{c}\right]}^{T}\in R^{C\times N}$. Then, we correlate $F_{r}$ and its transpose as an attention map: namely,

(6) $$\begin{array}{c} M_{c}^{\prime }=F_{r}F_{r}^{T} \end{array}$$

We further normalize $M_{c}^{\prime }$ using a softmax layer, i.e.,

(7) $$\begin{array}{c} M_{c}\left(i,j\right)=\frac{e^{M_{c}^{\prime }\left(i,j\right)}}{\sum_{j=1}^{C}e^{M_{c}^{\prime }\left(i,j\right)}}=\frac{e^{f_{i}\cdot f_{j}}}{\sum_{j=1}^{C}e^{f_{i}\cdot f_{j}}} \end{array}$$

Each element of $M_{c}\left(i,j\right)$ measures the similarity between the *i*th and *j*th channels and indicates the impact of channel *j* on channel *i*.

In order to guide the network to focus on discriminative regions, we further multiply $M_{c}$ and $F_{r}$ and reshape the result. The final output $F^{\prime }\in R^{C\times H\times W}$ is acquired by multiplying the refined output by a learnable parameter γ and performing an element-wise summation operation with F:

(8) $$\begin{array}{c} F^{\prime }=\gamma \cdot \mathrm{reshape}\left(M_{c}\cdot F_{r}\right)+F \end{array}$$

where γ is initialized to be 0 and gradually learns to assign more weight [36]. Equation (8) indicates that the refined feature map $F^{\prime }$ of each channel is a weighted summation of all channel features with the original features. Through the CAM, semantically significant parts of the feature map can be highlighted by integrating spatially correlated channels.

**Position Attention Module:** Discriminant feature representations are essential for fine-grained recognition; they can be obtained by capturing long-range contextual information. However, convolution mainly deals with local neighborhoods and must be stacked with many layers to (capture long-range dependencies to) associate different parts of the whole image. This introduces computational inefficiency and high complexity [37]. We use a position attention module (PAM) to mine locally discriminative regions. It can capture long-range dependencies directly by computing interactions between any two positions regardless of their positional distances.

The PAM takes the refined output $F^{\prime }$ of the CAM as input. Different from the CAM, the PAM first feeds $F^{\prime }$ into a convolutional layer with a kernel size of one to reduce parameter overhead. This generates two new feature maps: K and Q. The subsequent operations are similar to those for the CAM. The two feature maps are further reshaped to $\left\{K,Q\right\}\in R^{C\times N}$, where N=H×W is the number of pixels.

Then, we multiply the transpose of $K=\left[k_{1},k_{2},\ldots ,k_{N}\right]\in R^{C\times N}$ and $Q=\left[q_{1},q_{2},\ldots ,q_{N}\right]\in R^{C\times N}$ to obtain the attention map $M_{p}^{\prime }\in R^{N\times N}$:

(9) $$\begin{array}{c} M_{p}^{\prime }=K^{T}Q \end{array}$$

We further normalize $M_{p}^{\prime }$ by using a softmax layer:

(10) $$\begin{array}{c} M_{p}\left(i,j\right)=\frac{e^{M_{p}^{\prime }\left(i,j\right)}}{\sum_{i=1}^{C}e^{M_{p}^{\prime }\left(i,j\right)}}=\frac{e^{k_{i}\cdot q_{j}}}{\sum_{i=1}^{C}e^{k_{i}\cdot q_{j}}} \end{array}$$

where $M_{p}\left(i,j\right)$ measures the *j*th position’s impact on the *i*th position. Equation (10) indicates that any two pixels in the image can interact with each other, and space dependencies can directly be captured in a feedforward fashion by this method.

Meanwhile, in the same way that $\left\{K,Q\right\}$ is obtained, we feed the feature map $F^{\prime }$ into a convolution layer with a kernel size of one to generate a new feature map $V\in R^{C\times H\times W}$ and reshape it to $V\in R^{C\times N}$. Then, we multiply V and $M_{p}$ and reshape the result to $R^{C\times H\times W}$. The final output $F^{\prime \prime }\in R^{C\times H\times W}$ is achieved by multiplying the refined result by a learnable parameter δ and performing an element-wise summation with the original input feature map $F^{\prime }$:

(11) $$\begin{array}{c} F^{\prime \prime }=\delta \cdot \mathrm{reshape}\left(V\cdot M_{p}\right)+F^{\prime } \end{array}$$

where δ also starts from 0 and gradually learns a weight. An initialization of 0 means that the PAM is not used when the training starts; this is because the network is expected to learn the local information first. As training proceeds, the network will slowly try to use the PAM to learn the long-range dependencies between distant pixels.

Equation (11) indicates that each position of $F^{\prime \prime }$ is a weighted summation of features across all positions and the original features. PAM can selectively aggregate semantically similar parts in space, and discriminative regions achieve higher gains: thus facilitating fine-grained identification.

### 4.4. ASFFM

Previous works [38,39,40] have typically exploited element-wise summation or concatenation to fuse multi-scale features, which means that semantically strong parts are weighted the same as semantically weak parts. Inspired by [41], an ASFFM is proposed to adaptively learn the spatial weight of fusion for feature maps at each scale.

**Figure 4:**
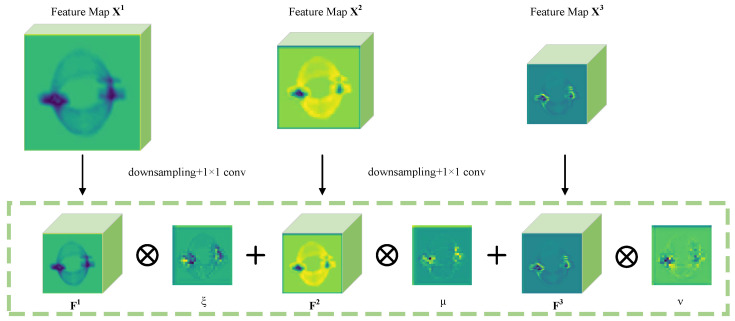
The structure of ASFFM.

As illustrated in Figure 4, let $X^{1}$, $X^{2}$, and $X^{3}$ denote the three input feature maps, respectively, among which, the resolution of the former is twice that of the latter, while the channel number of the former is 1/2 that of the latter. Thus, we first resize the feature maps to the same shape of $X^{3}$. Concretely, $X^{2}$ is fed into a 3×3 convolution layer with a stride of two to simultaneously modify the channel number and downsample the resolution to one-half of the original. For $X^{1}$, to achieve downsampling with a 1/4 ratio, we add a max-pooling layer with a stride of two before the two-stride convolution.

Let $F^{1}$, $F^{2}$, and $F^{3}$ denote the resized feature maps with the same size. Then, the three feature maps at different scales are integrated as follows:

(12) $$\begin{array}{c} F_{ij}^{f}=\xi_{ij}\cdot F_{ij}^{1}+\mu_{ij}\cdot F_{ij}^{2}+\nu_{ij}\cdot F_{ij}^{3} \end{array}$$

where $F_{ij}^{f}$ and $F_{ij}^{n}$, $n=\left\{1,2,3\right\}$, denote the descriptor at position $\left(i,j\right)$ on the feature maps; and $\xi_{ij}$, $\mu_{ij}$, and $\nu_{ij}$ are weights that indicate the importance of different positions on the feature map. These weights are adaptively learned by the network and are broadcast along the channel dimension during multiplication. Specifically, $\xi_{ij},\mu_{ij},\nu_{ij}\in \left[0,1\right]$ are formulated by a softmax layer:

(13) $$\begin{array}{cc} \xi_{ij} & =\frac{e^{\eta_{\xi_{ij}}}}{e^{\eta_{\xi_{ij}}}+e^{\eta_{\mu_{ij}}}+e^{\eta_{\nu_{ij}}}}\\ \mu_{ij} & =\frac{e^{\eta_{\mu_{ij}}}}{e^{\eta_{\xi_{ij}}}+e^{\eta_{\mu_{ij}}}+e^{\eta_{\nu_{ij}}}}\\ \nu_{ij} & =\frac{e^{\eta_{\nu_{ij}}}}{e^{\eta_{\xi_{ij}}}+e^{\eta_{\mu_{ij}}}+e^{\eta_{\nu_{ij}}}} \end{array}$$

where $\eta_{\xi }$, $\eta_{\mu }$, and $\eta_{\nu }$ are generated by applying a 1 × 1 convolution layer on $F^{1}$, $F^{2}$, and $F^{3}$, respectively, and thus, they can be learned through standard back-propagation.

## 5. Experiments

### 5.1. Dataset

To assess the performance of our proposed FGRFNet for learning discriminative parts and fine-grained image recognition, we conduct experiments on ADS-B, Zigbee, and LoRa datasets. Instead of the original time-domain RF signals, we utilized their DCTF representations with the parameters λ, ε, and *N*, which are described in Section 3.2 to be 10, 2, and 65, respectively.

**ADS-B:** An ADS-B signal contains geo-coordinates, velocities, altitudes, headings, as well as the unique identifier information of the commercial aircraft. They are easy to receive and decode but are subject to identity spoofing attacks. We used the public dataset found in [42], for which the raw I/Q data of the signals were obtained at a sampling rate of 8 MHz and with a 1090 MHz center frequency; we generated 40,000 DCTFs in 100 subordinate categories and included 32,000 images for training and 8000 images for testing.

**Zigbee:** A Zigbee signal is modulated with O-QPSK modulation according to the IEEE 802.15.4 standard [43]. In the public dataset [33], the receiver works at 10 MSPS (million samples per second), and for each Zigbee device, 5 measurements with 9 segments of 40 K samples are recorded. To fully evaluate the proposed method, we also added additive Gaussian white noise (AWGN) with different signal-to-noise ratios (SNRs) ranging from 0 to 25 dB to the received signals. We converted the received signals to DCTFs and obtained 24,300 DCTFs of 54 classes in total; 60% (14,580) of the images are used for training, and the remaining 40% (9720) are used for testing.

**LoRa:** LoRa technology is a wireless communication technology widely utilized by IoT applications. The publicly available LoRa dataset [44] was collected from 25 distinct IoT devices with LoRa functionality using a USRP B210 receiver. The dataset offers a comprehensive collection of fundamental experimental scenarios, including indoor and outdoor environments as well as various network deployments and configurations. For each class, we partitioned 320 samples for training and 80 samples for testing.

### 5.2. Implementation

In the training stage, we first normalize the input DCFTs. Then, we use either ResNet-50 or ResNet-101 as the backbone and choose the last output features of the residual blocks from Stage 3, Stage 4, and Stage 5 in ResNet to establish a pyramidal hierarchy. We take a tensor of size [3, 64, 64] as an example, where the first number in the tensor represents the channels, and the subsequent two numbers respectively represent the height and width of the image. As the features pass through each stage, the number of channels doubles, and the size reduces by half. Consequently, the feature sizes of the outputs for Stages 3, 4, and 5 are [512, 8, 8], [1024, 4, 4], and [2048, 2, 2], respectively. We take the output of Stage 3 as an example to analyze how the CPAM and ASFFM operate.

Suppose the tensor size of F is [512, 8, 8]. First, channel attention is performed by reshaping F into [512, 16], denoted as $F_{r}$. After the covariance operation, the channel attention matrix $M_{c}^{\prime }$ has a size of [512, 512]. Normalizing it with a softmax layer yields attention scores $M_{c}$ of size [512, 512]. Multiplying the attention scores by $F_{r}$ and reshaping the result into [512, 8, 8] followed by multiplication with a learnable parameter and addition to the original input feature map gives the refined feature map after channel attention mechanism correction, denoted as $F^{\prime }$ and with a size of [512, 8, 8]. Spatial attention calculation follows a similar process. Using the output $F^{\prime }$ of channel attention as input, three 1×1 convolutions are applied to obtain K, Q, and V with a size of [512, 8, 8]. After reshaping Q and K into [512, 16], multiplication is performed, and normalization yields spatial attention scores $M_{p}$ with a size of [16, 16]. Multiplying the attention scores by V, reshaping the result into [512, 8, 8], and calculating based on Equation (11) gives the feature map $F^{\prime \prime }$ after the attention mechanism.

From the above analysis, it is evident that the size of the feature map remains unchanged after the CPAM. Therefore, the feature-enhanced outputs $X^{1}$, $X^{2}$, and $X^{3}$ of Stages 3, 4, and 5 remain [512, 8, 8], [1024, 4, 4], and [2048, 2, 2], respectively. Subsequently, the ASFFM integrates these features. After applying max-pooling layers or two-stride convolution layers to resize $X^{1}$ and $X^{2}$ to [512, 8, 8], denoted as $F^{1}$, $F^{2}$, respectively, 1 × 1 convolutions are applied to $F^{1}$, $F^{2}$, and $F^{3}$ to obtain fusion weights. Multiplying the feature maps by their respective fusion weights results in the size of the adaptively spatially fused feature being [512, 8, 8]. This is then fed into the classifier for experimental radio frequency identification.

During training, the learning rate is initially set to 0.005 with a series of decays to change the value to one-tenth of the original value per 10 epochs. The weight decay is set to 0.0005, the stochastic gradient descent (SGD) algorithm is used as the optimizer, and the batch size is set to 32. On the ADS-B dataset, 100 epochs are trained, and 80 epochs are trained on the Zigbee and LoRa datasets.

During testing, we evaluate the performance of RF fingerprint recognition using metrics such as accuracy, F1-score, and AUC (Area Under the ROC Curve). F1-score combines precision and recall into a single value, providing a balanced measure of a classifier’s accuracy. The higher the three metrics mentioned above, the better the classification performance.

All experiments are carried out on a single Nvidia GeForce RTX 3090 GPU card, and the PyTorch toolbox is used as the main implementation substrate.

**Figure 5:**
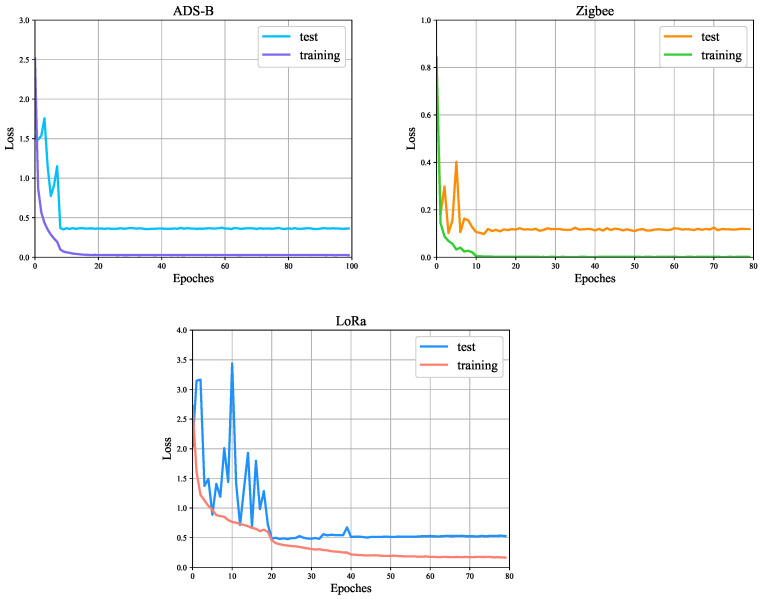
Training loss and test loss of the FGRFNet on the ADS-B, Zigbee, and LoRa datasets.

Figure 5 shows the training and test loss curves of the FGRFNet on the three datasets. As can be seen, the training loss reaches a significantly low value, and this indicates that the model fits the data well.

### 5.3. Comparison and Discussion

To further validate the proposed method, we compare it with other state-of-the-art methods for both fine-grained recognition and specific emitter identification fields in Table 1 and Table 2. In Table 1, our FGRFNet achieves a significant improvement over the corresponding backbones on the ADS-B dataset, with clear margins of 4.21% and 3.66% in accuracy on ResNet-50 and ResNet-101, respectively. In terms of F1-score and AUC, our network achieved the highest values of 0.8982 and 0.9989, respectively, surpassing the other methods. This suggests strong discriminatory ability and reliability of the network. Compared with ICAM [45], which integrates a plug-and-play attention block into the backbone to enhance the exploitation of spatial and channel information by the convolutional layers, we get a better result, with a relative accuracy improvement of 1.83%. In comparison to methods that solely utilize a few simple convolutional layers, such as AlexNet [46] and CNN [33], our approach yields superior results, with a notable relative accuracy improvement of over 6.5%. This shows the superior attention learning ability of the proposed approach. In addition, compared with the leading result achieved by GoogLenet, our method has an improved accuracy of 1.04% with ResNet-50.

Table 2 lists several representative approaches with high accuracy on the Zigbee and LoRa datasets. On both datasets, our network achieves AUC values of 0.9999 and 0.9915, respectively, indicating that our network is very close to being a “perfect classifier”. Using ResNet-50 as the baseline, our method still achieves a competitive accuracy of 83.00%, which is 2.55% and 4.2% better than MobileNetV3 [49] and GoogLenet [46], respectively, on the LoRa dataset. ICAM [45] and ARFNet [19] also utilize spatial and channel attention mechanisms. However, our approach outperforms them on the Lora dataset with improvements of over 2% in accuracy and F1-score. In contrast to the attention mechanisms employed in the aforementioned two methods, our CPAM facilitates interactions between every pixel, rather than confining interactions solely to local regions as in ICAM and ARFNet, providing a more robust modeling capability. This empowers the model to better grasp long-range dependencies between elements. Furthermore, our method ensures precise attention scores at each pixel for positional attention, facilitating the extraction of finer features and capturing subtle differences between different devices, thereby enabling effective discrimination between them. Additionally, FGRFNet incorporates ASFFM to fuse features from different scales, merging high-level semantic features with low-level details. This allows the model to extract more discriminative fine-grained features. Consequently, our approach outperforms other state-of-the-art methods and achieves the highest scores across all three metrics.

### 5.4. Ablation Study

To analyze the contributions of different components in the proposed framework, we conduct various ablation experiments on the three datasets.

**Impact of CPAM:** In Table 3, compared with the backbone-only model, the CPAM-based model enhances the accuracy by 2.81% for ADS-B, 0.77% for Zigbee, and 3.7% for LoRa. This shows that CPAM can localize the discriminative parts to learn the fine-grained part-feature representation, which is beneficial for fine-grained RF fingerprint recognition.

Table 3 also presents the contributions of CAM and PAM to the entire attention module. CAM calculates the importance of each channel of the input image and thus makes the input image more meaningful. That is, a channel with key information will be given a higher weight, and one without important information will be given a lower weight. In this way, more informative discriminative regions can be located, and background noise can be excluded. Meanwhile, PAM can capture long-distance spatially related information and make up for the limited receptive field of CNN (owing to the CNN’s limited convolution kernel size). The combination of CAM and PAM enables CPAM to locate key areas more accurately, which is helpful for learning region-based fine-grained features.

**Impact of ASFFM:** In Table 4, with the ASFFM, the backbone model and the backbone–CPAM model achieve accuracy improvements of 2.43% and 1.78%, respectively, on the ADS-B dataset. This indicates that the ASFFM can integrate low-level detailed information with enhanced high-level semantic information features and thus learn part-feature representations for fine-grained classification by fusing pyramidal features.

### 5.5. Impact of DCTF Generation Parameters

DCTF image generation is influenced by various factors, including differential interval λ, I/Q phase distortion ε, DCTF sizes, and sample lengths. DCTF exhibits significant variations under different differential intervals and I/Q phase mismatches. Therefore, it is essential to search for optimal parameters to maximize the distinction between different devices. At the same time, image sizes and the number of samples also affect the quality of DCTF. Larger image sizes decrease the likelihood of different samples coinciding within the same pixel; this necessitates a higher number of samples to preserve graphic details. Conversely, smaller image sizes result in blurred features between devices. Therefore, in this section, we investigate the effect of the DCTF generation parameters on the performance of the model using the Zigbee dataset.

**Figure 6:**
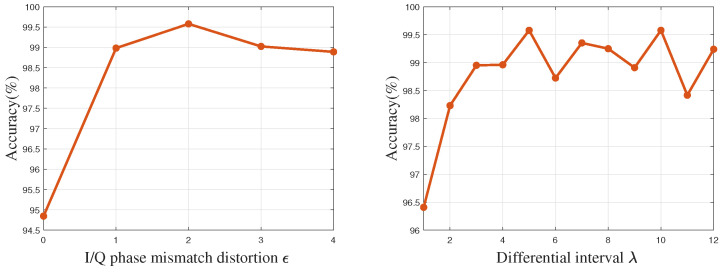
Accuracy with different generation parameters.

Figure 6 illustrates the accuracy of FGRFNet under various DCTF generation parameters when the SNR is 25 dB and the DCTF size is 64×64. As shown in Figure 6, the left plot demonstrates the impact of varying ε on the accuracy of the model when λ is fixed at 10. It can be observed that setting ε to 0 leads to a significant decrease in accuracy, while for ε greater than 1, the overall accuracy consistently remains above 98%. Particularly, the best performance is achieved when ε is set to 2. The right plot depicts the influence of different λ values on the model’s accuracy when ε is held constant at 2. It is evident that setting extremely short differential intervals leads to a significant drop in accuracy. However, when λ exceeds 3, the overall accuracy consistently surpasses 98.5%. Notably, the optimal performance is observed when λ is set to 10.

**Figure 7:**
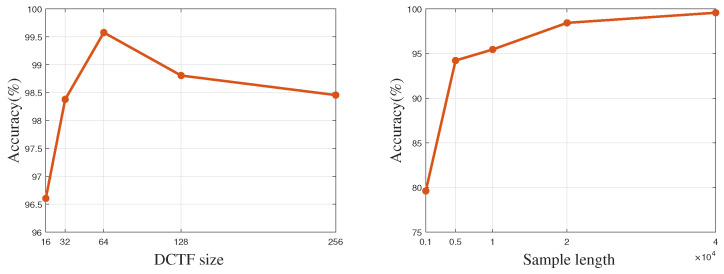
Accuracy with different data sizes and sample lengths.

The accuracy when using different DCTF image qualities is illustrated in Figure 7 for an SNR of 25 dB, λ=10, and ε=2. The left graph presents the relationship between model accuracy and DCTF sizes under a fixed sample length. It is evident that when the image size is too small, such as below 16×16, the RF fingerprint features become indistinct, resulting in a decline in model accuracy. However, as the image size decreases, the computational complexity of the network also decreases. Experimental results indicate that an image size of 64×64 provides the best performance of FGRFNet with relatively low complexity. With a fixed DCTF size of 64×64, the right graph clearly illustrates that as the number of samples used for generating DCTF increases, the quality of the DCTF images improves, resulting in enhanced recognition accuracy.

### 5.6. Impact of Distance

Table 5 describes the impact of the distance between the transmitter and receiver on recognition accuracy. The study examines two specific distances: 5 m and 15 m. For each distance, data from 25 different LoRa devices were collected and analyzed. It is evident that the recognition accuracy of the model decreases as the distance between the transmitter and receiver increases, owing to the impact of varying channel conditions. Notably, methods like CNN [33] and AlexNet [46] exhibit relatively lower precision. Thus, we can infer that varying channel conditions have a significant impact on the accuracy of CNN and AlexNet methods. The experimental results indicate that our proposed FGRFNet exhibits a certain level of resilience against channel effects, showcasing more stable feature extraction compared to other methods. As the distance increases and the SNR decreases, the accuracy of this approach gradually declines.

### 5.7. Impact of SNR

**Figure 8:**
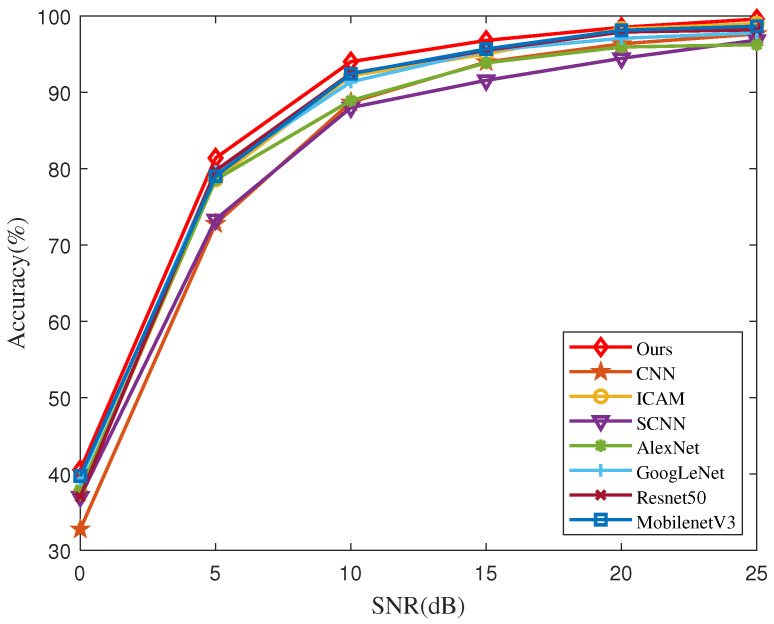
Accuracy of CNN, SCNN, AlexNet, GoogLenet, ResNet-50, ICAM, MobileNetV3, and FGRFNet on the Zigbee dataset for different SNRs.

**Figure 9:**
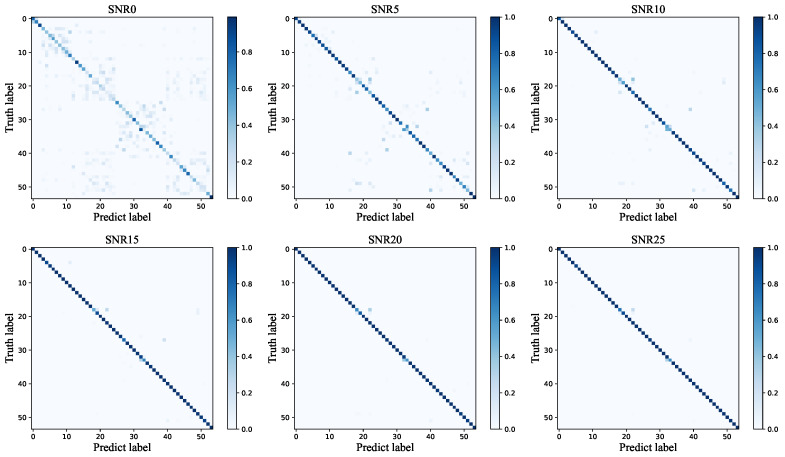
Confusion matrix of FGRFNet on the Zigbee dataset for different SNRs.

In order to evaluate the influence of SNR on RF fingerprinting recognition, we also carried out experiments under different SNR levels in the range of [0, 25] dB at a step of 5 dB using the Zigbee dataset. Figure 8 draws the accuracy curves using CNN [33], SCNN [51], AlexNet [46], GoogLenet [46], ResNet-50 [25], ICAM [45], MobileNetV3 [49], and our method for different SNR levels. It can be seen that as the SNR decreases, all the accuracies degrade. The performance deteriorates substantially, especially when the SNR is lower than 15 dB. But FGRFNet achieves an overall higher accuracy than other methods. In particular, when the SNR is lower than 15 dB, FGRFNet exhibits a performance advantage of approximately 10% over CNN. This indicates that FGRFNet is capable of effectively learning discriminative features and exhibiting greater robustness to noise. Figure 9 further displays the recognition confusion matrix of FGRFNet for different SNRs. With increasing SNR, the accuracy of RF fingerprint recognition is improved as well.

### 5.8. Visualization

We adopt gradient-weighted class activation mapping (Grad-CAM) [52] to visualize and analyze the learned RF fingerprints to make our FGRFNet more interpretable. Grad-CAM generates heat maps that show the contribution distribution of the predicted output for input images. A higher score indicates a stronger response and greater contribution from the corresponding areas of the original image. This highlights the importance of each location to the class.

**Figure 10:**
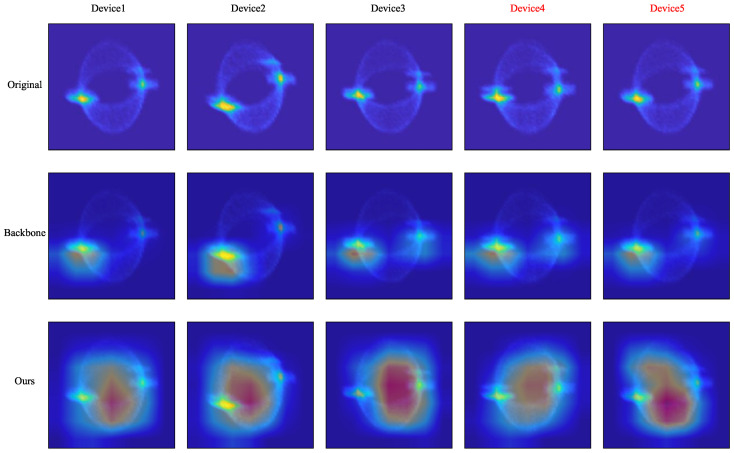
Visualization of different Zigbee devices using Grad-CAM.

We selected five DCTFs from five different Zigbee devices at 25 dB for visualization. Figure 10 displays the original DCTFs along with the visualization results of the backbone and FGRFNet. Device 4 and Device 5 are misclassified by the backbone ResNet but are correctly classified by FGRFNet. Through careful observation, we can see that FGRFNet pays more attention to the global structure, while the backbone ResNet just focuses on the gathering centers. As pointed out in Section 3.2, the RF fingerprint is related to the locations of gathering centers as well as the deviation and dispersion of constellation points. Locations of gathering centers effectively reflect the inherent defects in the transmitter brought about by the I/Q modulator, such as I/Q gain imbalances, DC offsets, and CFO. In addition, constellation point deviation and dispersion suggest the inherent non-linear characteristics of the power amplifier.

Figure 10 demonstrates that the backbone network selectively focuses on prominent gathering centers, indicating that it solely learns the inherent defects of the transmitter caused by the I/Q modulator, such as I/Q gain imbalances, DC offsets, and carrier frequency offsets. However, FGRFNet captures additional subtle and fine-grained features, including constellation point deviation and dispersion, indicating that FGRFNet simultaneously learns the non-linearities of the amplifier and the inherent defects of the transmitter caused by the I/Q modulator: namely, the RF fingerprints. The proposed FGRFNet successfully learns more implicit and fine-grained features, such as the non-linearities of the amplifier, thereby achieving higher performance in Figure 10.

## 6. Conclusions

In this article, we developed a fine-grained RF-fingerprint-based recognition network to identify different wireless devices by fusing multi-level features that are enhanced by CPAMs to deliver both the high-level semantic and the low-level detailed information. The attention module can effectively locate discriminative regions and reduce the effect of background noise, and the fusion module can enhance the recognition robustness. Experimental results on the ADS-B, Zigbee, and LoRa datasets verified the effectiveness of the proposed FGRFNet.

While our method achieved excellent performance, it comes with some drawbacks. On the one hand, there is relatively high computational complexity, primarily due to covariance calculations in the attention module, leading to a substantial increase in the number of parameters. On the other hand, this method is only suitable for closed-set classification: meaning that the device categories in the training set and the test set must be the same.

However, in real-world scenarios, the electromagnetic environment is open and dynamic, and the types and quantities of wireless devices can vary and remain uncertain. The closed-set authentication approach may not be suitable for handling such situations. Therefore, in addition to advancing fine-grained recognition techniques, we also aim to explore open-set recognition methods in the future. By dedicating our efforts to open-set recognition, we aim to address the challenges posed by the dynamic and diverse nature of the IoT environment. Our ultimate goal is to facilitate the widespread and practical application of RF fingerprint recognition in IoT settings.

## Figures and Tables

**Table 1:** Comparison of different methods on the ADS-B dataset.

Method	Backbone	Accuracy (%)	F1-Score	AUC
PMG [47]	-	79.18	0.7891	0.9734
CNN [33]	-	79.37	0.7905	0.9866
AlexNet [46]	-	82.35	0.8225	0.9837
API-Net [29]	ResNet-101	82.73	0.8192	0.9879
CSIL [48]	-	83.50	0.8324	0.9882
ACNet [30]	ResNet-50	84.41	0.8326	0.9881
MobileNetV3-Large [49]	-	85.29	0.8516	0.9884
ResNet-50 [25]	-	85.03	0.8480	0.9896
ResNet-101 [25]	-	86.20	0.8603	0.9899
SEF [50]	ResNet-50	86.99	0.8682	0.9923
ICAM [45]	ResNet-50	87.41	0.8732	0.9925
GoogLenet [46]	-	87.76	0.8767	0.9957
ours	ResNet-50	**89.24**	**0.8913**	**0.9984**
ours	ResNet-101	**89.86**	**0.8982**	**0.9989**

**Table 2:** Comparison of different methods on the Zigbee and LoRa datasets.

Method	Backbone	Accuracy (%)		F1-Score		AUC	
		Zigbee	LoRa	Zigbee	LoRa	Zigbee	LoRa
CNN [33]	-	97.63	67.20	0.9759	0.6673	0.9996	0.9633
SCNN [51]	-	96.83	76.45	0.9663	0.7611	0.9997	0.9845
AlexNet [46]	-	96.22	61.65	0.9590	0.5981	0.9993	0.9569
GoogLenet [46]	-	97.78	78.80	0.9758	0.7846	0.9994	0.9821
ResNet-50 [25]	-	98.16	78.35	0.9812	0.7813	0.9996	0.9855
ICAM [45]	ResNet-50	98.99	81.40	0.9898	0.8104	0.9997	0.9883
MobileNetV3 [49]	-	98.64	80.45	0.9863	0.8051	0.9997	0.9886
ARFNet [19]	ResNet-50	98.35	79.41	0.9830	0.9996	0.7929	0.9865
ours	ResNet-50	**99.57**	**83.00**	**0.9957**	**0.8294**	**0.9999**	**0.9915**

**Table 3:** Ablation study of the attention module.

Method	Accuracy (%)		
	ADS-B	Zigbee	LoRa
Backbone	85.03	98.16	78.35
Backbone + CAM	87.14	98.26	81.10
Backbone + PAM	86.26	98.30	80.75
Backbone + CPAM	87.84	98.93	82.05

**Table 4:** Ablation study of our method.

Method	Accuracy (%)		
	ADS-B	Zigbee	LoRa
Backbone	85.03	98.16	78.35
Backbone + CPAM	87.84	98.93	82.05
Backbone + ASFFM	87.46	98.57	81.55
Backbone + CPAM + ASFFM	89.24	99.57	83.00

**Table 5:** Accuracy comparison when varying the distance between the transmitters and the receiver.

Method	Accuracy (%)	
	5 m	15 m
CNN [33]	60.80	33.05
SCNN [51]	62.55	33.60
AlexNet [46]	57.25	33.80
GoogLenet [46]	62.05	37.10
ResNet-50 [25]	66.90	37.70
ICAM [45]	66.20	33.75
MobileNetV3 [49]	66.35	37.50
ours	**68.40**	**39.30**

## Data Availability

Our source codes have been released at https://github.com/zhangyu1an/FGRFNet (accessed on 22 December 2023).

## Abbreviations

The following abbreviations are used in this manuscript:

FGRFNet	Fine-grained RF Fingerprint Recognition Network
DCTF	Differential Constellation Trace Figure
CFO	Carrier Frequency Offset
ICAO	International Civil Aviation Organization
CPAM	Channel and Positional Attention Module
ASFFM	Adaptive Spatial Feature Fusion Module
